# *De Novo* Transcriptome Sequencing Analysis of Goose (*Anser anser*) Embryonic Skin and the Identification of Genes Related to Feather Follicle Morphogenesis at Three Stages of Development

**DOI:** 10.3390/ijms19103170

**Published:** 2018-10-15

**Authors:** Chang Liu, Cornelius Tlotliso Sello, Yongfeng Sun, Yuxuan Zhou, Hongtao Lu, Yujian Sui, Jingtao Hu, Chenguang Xu, Yue Sun, Jing Liu, Shengyi Li, Yiming Zhang, Kaiyan Zhang

**Affiliations:** 1 College of Animal Science and Technology, Jilin Agricultural University, Changchun 130118, China; xinrongjielu@163.com (C.L.); sello@jlau.edu.cn (C.T.S.); joe199697@163.com (Y.Z.); luhongtao4755@163.com (H.L.); suiyujian2000@163.com (Y.S.); hujingtao2004@126.com (J.H.); x924299@163.com (C.X.); ningxin20121216@163.com (Y.S.); L1145545376@163.com (J.L.); 18104476170@163.com (S.L.); z15584002809@163.com (Y.Z.); 17843096083@163.com (K.Z.); 2 Key Laboratory for Animal Production, Product Quality and Safety of Ministry of Education, Changchun 130118, China

**Keywords:** feather follicle development, *de novo* transcriptome assembly, cluster analysis, differentially expressed genes

## Abstract

The objective of this study was to evaluate the changes in the goose embryo transcriptome during feather development. RNA-Sequencing (RNA-Seq) was used to find the transcriptome profiles of feather follicles from three stages of embryonic dorsal skin at embryonic day 13, 18, and 28 (E13, E18, E28). The results showed that 3001, 6634, and 13,780 genes were differently expressed in three stages. Gene Ontology (GO) and Kyoto Encyclopedia of Genes and Genomes (KEGG) analysis revealed that differentially expressed genes (DEGs) in E13 vs. E18 were significantly mapped into the GO term of extracellular structure organization and the pathway of extracellular matrix (ECM)-receptor interaction. In E18 vs. E28, the top significantly mapped into GO term was the single-organism developmental process; the pathway was also the ECM-receptor interaction. DEGs in E13 vs. E28 were significantly mapped into the GO term of the multicellular organismal process and the pathway of cell adhesion molecules. Subsequently, the union of DEGs was categorized by succession cluster into eight profiles, which were then grouped into four ideal profiles. Lastly, the seven genes spatio-temporal expression pattern was confirmed by real-time PCR. Our findings advocate that interleukin 20 receptor subunit alpha (*IL20RA*), interleukin 6 receptor (*IL6R*), interleukin 1 receptor type 1 (*IL-1R1*), Wnt family member 3A (*WNT3A*), insulin-like growth factor binding protein 3 (*IGFBP3*), bone morphogenetic protein 7 (*BMP7*), and secreted-frizzled related protein 2 (*SFRP2*) might possibly play vital roles in skin and feather follicle development and growth processes.

## 1. Introduction

Annually comprehensively large amounts of feathers production are the by-product of poultry processing and have uses in light-weight structural materials, green house industry, artwork, paper alternatives, animal feeds as protein source, diaper filling, biodegradable composites, soil erosion control, fabric, water filtration fibers, upholstery, automotive industries, aircraft, and paper alternatives [1,2]. Goose feathers are highly profitable and more in particular downy feathers provide a good quality material for bedding and clothing conforming excellent insulation to retain warmth since they are bulky and light in weight [3]. Therefore, more attention in recent studies is directed towards the improvement of goose feather follicles. Feathers in various body regions have different forms in color, structure, branching patterns, and functional properties amongst different fowl species and within individual birds [4]. In structure, poultry feathers can mainly be divided into three parts: bilaterally symmetric contour feathers and bilaterally asymmetric flight feathers that are derived from primary feather follicles, and radially symmetrical downy feathers that are derived from secondary feather follicles [5,6]. The formation and regulation of feather follicles during embryogenesis require a sequence of sophisticated molecular intercommunication between the epithelium and mesenchyme, triggered by the first dermal message from the mesenchyme that promotes the establishment of placodes arrays, or thickenings, in the surface epithelium [7]. To exploit the commercial potential of poultry feathers, goose could be used as the evident model to acquire knowledge and understanding of molecular mechanisms regulating the early stages (primary and secondary follicles) of feather development. 

Gene regulation is the basis for all biological behaviours and phenotypes, and multiple proteins and growth factors, therefore the study of regulatory factors controlling genetic information still remain as the challenge in molecular researches [8]. Multiple transcription factors in higher organisms play an essential role by controlling gene expression regulatory factors that trigger growth, development, and evolution [9]. Up to date, the regulation of molecular mechanisms on feather follicles formation and development has been reported in several relevant types of researches in chicken [10,11,12,13] and duck [14]. Hence, studying the transcriptome profile of the goose embryonic feather follicle development would pave the way towards improving the quality and production of down feathers. RNA-Sequencing (RNA-Seq) employs high-throughput sequencing technologies to identify the structure, expression and function of genes at the integral level through the Illumina HiSeq 4000 sequencing platform [15]. *Anser anser* goose belongs to a non-model animal without a reference genome, but we can use *de novo* sequencing and bioinformatics software to assemble sequence regarding gaining the comprehensive genomic information [16]. In this study, we firstly observed the growth and development of embryonic dorsal skin in goose physically divided into three stages (the primordial period of primary feather follicles, the primordial period of secondary feather follicles, and the greater developmental period of secondary feather follicles) by histological observation. Then, we used *de novo* RNA-sequencing to compare the transcriptome profiles of feather follicles at different developmental stages, examining the concomitant transcriptional genes coordinating the substantial processes. The obtained differentially expressed genes (DEGs) at each stage in the current study will help the forthcoming investigation on the regulatory mechanisms in the primary and secondary feather follicles, and make provision to insight for down-type goose breeding.

## 2. Results

### 2.1. Micro Anatomic Observation of Feather Follicle Characters

**Figure 1:**
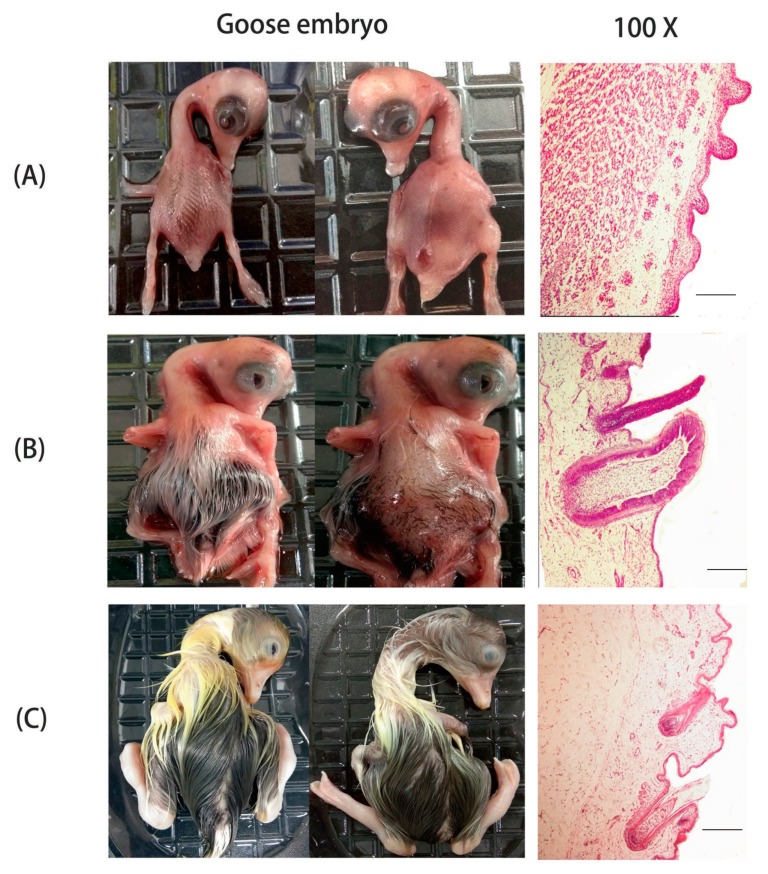
Three different stages in goose embryo skin development based on morphogenesis. Three different stages in goose embryo skin development were analyzed: (**A**) At embryonic day 13 (E13), primordial period of primary feather follicles. (**B**) At embryonic day 18 (E18), primordial period of secondary feather follicles. (**C**) At embryonic day 28 (E28), greater developmental period of secondary feather follicles. Histological sections of the three stages of goose skin during embryonic development (the first column and the second column: photograph; the third column: magnified 100×).

Cell proliferation in the feather bud epithelium was noticeable during the early stage of embryonic feather development. The dorsal tract buds extended at embryonic day 13 (E13) (Figure 1A), folding inwards to become feather follicles that were the blueprint of primary feather follicles. The diameter of the secondary feather follicles at embryonic day 18 (E18) was greatly smaller than that of primary feather follicles (Figure 1B). During the continuous inward folding and distal growth, the deep-narrow-pit shape was observed in the follicle and the feather germs resembled a long cylindrical shape piercing out of the follicles (Figure 1B). Therefore E18 was regarded as the primordial period of secondary feather follicles. At embryonic day 28 (E28), the follicle was filled completely with the feather bud, and the follicular cavity was disappearing, there was a close interconnection resulting into a single layer between feather and the follicle sheath. Also, the hair papilla was visible at the end of the follicle (Rugby-Ball shape) (Figure 1C).

### 2.2. Transcriptome Sequencing, De Novo Assembly, and Functional Annotation

To obtain a transcriptome reference for the goose, we used the RNA samples to construct RNA-seq libraries from three stages (E13: the primordial period of primary feather follicles; E18: the primordial period of secondary feather follicles; and, E28: the greater developmental period of secondary feather follicles). The aggregate of 485,026,174 raw reads were accumulated by using the Illumina HiSeq 4000 platform to do the paired-end sequencing of the nine constructed libraries. After filtering for the adaptor, assessment of contaminated ribosomal RNA (rRNA) and low-quality sequences, 473,955,926 qualified Illumina reads were obtained (Table 1), and the number of bases obtained by the clean reads was 69,782,488,597 nucleotides (nt). Subsequently, these high-quality cleaned reads from the nine individual libraries were *de novo* assembled into unique transcripts (unigenes). By using the Trinity package, 52,886 unigenes were assembled and at level N50, 2731 nt were obtained.

**Figure 2:**
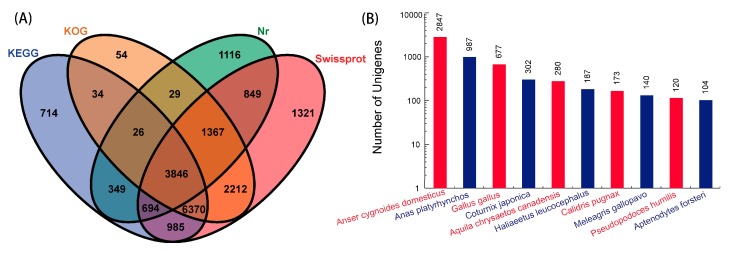
Characteristics of unigenes homology search in goose. (**A**) Venn diagram showed the number of unigenes annotated by BLASTx in different stages of feather follicle development, numbers in the circles indicate the number of unigenes annotated by single or multiple databases. (**B**) Number of unigenes matching to the 10 top species using BLASTx in the National Center for Biotechnology Information (NCBI) non-redundant (Nr) database.

To annotate the assembled sequences, the BLASTx comparison was used to align the unigene sequences with the protein databases National Center for Biotechnology Information (NCBI) non-redundant (Nr), Swiss-Prot, Kyoto Encyclopedia of Genes and Genomes (KEGG), and Eukaryotic Orthologous Groups (KOG), setting a cut-off *E*-value of 10^−5^. In summary, 52,886 unigenes were annotated after alignment with the four databases, while 8276 (15.65%) unigenes were annotated in the Nr database, 17,644 (33.36%) in the Swiss-Prot database; 13,018 (24.62%) in the KEGG database; and, 13,938 (26.35%) in the KOG database (Figure 2A). Based on the annotation results of the Nr database, 12.55% of the unigenes showed homology, the range of *E*-value was between 1 × 10^−20^ and 1 × 10^−5^; while 28.86% of those ranged between 1 × 10^−100^ and 1 × 10^−20^, and the remaining 58.59% of the unigenes had very strong homology with the *E*-value < 1 × 10^−100^ to available sequences. For species distribution, as the Nr database is shown in Figure 2B, a large number of unigenes in goose hit a wide range of animal species, including, *Gallus gallus*, *Anser cygnoides domesticus*, and *Anas platyrhynchos*. Among them, the largest number of homologous genes between *Anser anser* and *Anser cygnoides domesticus* suggests the close relationship between these two animals although there are differences in embryonic feather colouration. This may also advocate that *Anser cygnoides domesticus* transcriptomes and genomes could be used as a reference sequence for *Anser anser*. However, only 104 (0.20%) unigenes showed homology with sequences of *Aptenodytes forsteri*.

### 2.3. Differential Gene Expression at Embryonic Day 13, 18, and 28 (E13, E18, and E28)

To enhance the study on biological mechanisms of feather formation, the specific significance was the identification of DEGs at each stage. We obtained nine transcriptomes from different samples (E13, E18, and E28) and compared the transcription levels of each unigene between different samples. 3001 (2029 up-regulated and 972 down-regulated), 6634 (3662 up-regulated and 2972 down-regulated), and 13,780 (7862 up-regulated and 5918 down-regulated) DEGs were identified in E13 vs. E18, E18 vs. E28, and E13 vs. E28, respectively (Tables S1–S3).

Interleukins (ILs) are an essential multifunctional group of cytokines that mainly control the immune cell proliferation, growth, differentiation, and survival; however, their uncontrolled proliferation and interaction with non-immunity cells may be associated to organ damage risks [17,18]. Our analysis identified 29 DEGs belonging to common families of interleukins (ILs), and their target receptors (Table 2). Some of them were down-regulated in E13 vs. E18, E18 vs. E28, or E13 vs. E28, but the most were markedly up-regulated.

### 2.4. Gene Ontology (GO) Enrichment Analysis of Differentially Expressed Genes (DEGs)

**Figure 3:**
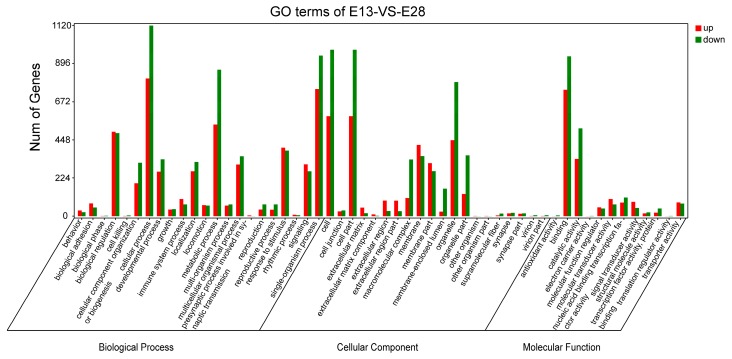
Gene Ontology (GO) classifications of DEGs among libraries in E13 vs. E28. The results are summarised in three main categories: biological process, cellular component, and molecular function. The *X*-axis indicates the secondary classification of hierarchical GO terms; The *Y*-axis shows the number of genes.

We used Gene Ontology (GO) enrichment analysis to classify gene functions of the DEGs identified, and DEGs were categorized into three GO groups: molecular function, cellular component, and biological process. The DEGs in E13 vs. E18 were significantly enriched (*p* < 0.05) in 428 molecular functions, 271 cellular components, and 2543 biological processes, respectively (Table S4). The DEGs in E18 vs. E28 showed significant enrichment in 322 cellular component, 2976 biological process, and 514 molecular functions, respectively (Table S5). The DEGs in E13 vs. E28 showed significant enrichment in 404 cellular component, 3639 biological processes, and 687 molecular functions, respectively (Table S6). In E13 vs. E18, E18 vs. E28, and E13 vs. E28, the top five functional categories of up-regulation of DEGs were in the same order, including cellular process, single-organism process, binding, cell, and cell part. For the cellular component category, it was noteworthy that large numbers of up-regulation of DEGs, as well as down-regulation of DEGs were categorized as cell part, cell, and organelle in E13 vs. E18, E18 vs. E28, and E13 vs. E28. For the biological process, the large numbers of DEGs were categorized as the cellular process, single-organism process, and metabolic process in E13 vs. E18, E18 vs. E28, and E13 vs. E28. For the molecular function, the large numbers of DEGs were categorized as binding, catalytic activity, and nucleic acid binding transcription factor activity in E13 vs. E18, E18 vs. E28, and E13 vs. E28 (Figure 3). Further enrichment analysis was found to be related to the developmental process, and the detected DEGs were enriched in different terms, such as *WNT3A* (Wnt family member 3A), *IGFBP3* (insulin-like growth factor binding protein 3), *BMP7* (bone morphogenetic protein 7), and *SFRP2* (secreted-frizzled related protein 2).

### 2.5. Kyoto Encyclopedia of Genes and Genomes (KEGG) Pathway Enrichment Analysis of DEGs

**Figure 4:**
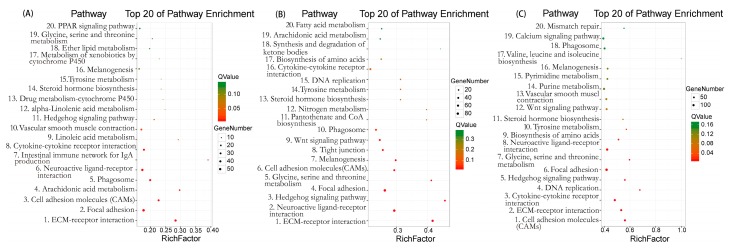
Top 20 pathway enrichment in Kyoto Encyclopedia of Genes and Genomes (KEGG) pathway analysis. (**A**) E13 vs. E18. (**B**) E18 vs. E28. (**C**) E13 vs. E28. The greater the Rich Factor, the higher the degree of enrichment. The *Q* value ranges from 0 to 1 and the closer it is to zero, the more significant the enrichment.

In the present study, we did the pathway analysis of DEGs by using the KEGG database. In E13 vs. E18, there were 592 DEGs mapped into 156 KEGG pathways (Table S7), the top 20 enriched pathways are shown in Figure 4A, and the top five significantly mapped KEGG pathways were extracellular matrix (ECM)-receptor interaction (ko04512), focal adhesion (ko04510), cell adhesion molecules (CAMs) (ko04514), arachidonic acid metabolism (ko00590), and phagosome (ko04145). The DEGs in E18 vs. E28 were mapped into 193 KEGG pathways (Table S8), the top 20 enriched pathways are shown in Figure 4B and the top five KEGG pathways showing the highest demonstration of the DEGs were ECM-receptor interaction (ko04512), neuroactive ligand-receptor interaction (ko04080), Hedgehog signaling pathway (ko04340), focal adhesion (ko04510), and glycine, serine, and threonine metabolism (ko00260). The most significantly mapped pathway is also the ECM-receptor interaction. In E13 vs. E28, the DEGs were mapped into 202 KEGG pathways (Table S9), the maps with the highest unigene representation were cell adhesion molecules (CAMs) (ko04514) with 79 (3.92%) candidate genes, followed by ECM-receptor interaction (ko04512), cytokine-cytokine receptor interaction (ko04060), DNA replication (ko03030), and Hedgehog signaling pathway (ko04340). The top 20 enriched pathways are shown in Figure 4C.

### 2.6. Cluster Analysis of DEGs among the Three Developmental Stages

**Figure 5:**
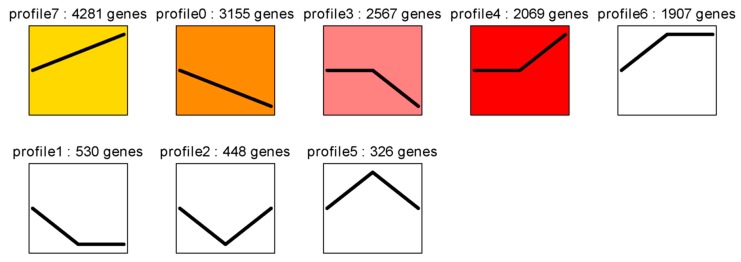
Eight profiles of DEGs with unique expression alterations over E13, E18, and E28. The profile number and the number of genes are shown on top of each square. The number of genes assigned is used to order the profiles. The profiles with color (*p* < 0.05): significant enrichment trend. The profiles without color: non-significant enrichment trend.

**Figure 6:**
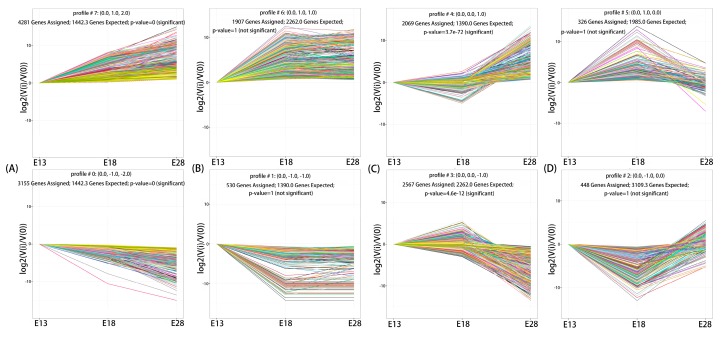
The four cluster trajectory profiles with two antagonistic profiles of DEGs. (**A**) Model profile A, including profiles #7 and #0. (**B**) Model profile B, including profiles #6 and #1. (**C**) Model profile C, including profiles #4 and #3. (**D**) Model profile D, including profiles #5 and #2. Each *X*-axis indicates the feather follicles development state (E13, E18, and E28); *Y*-axis shows expression changes.

To determine the gene expression trajectories, we used the Science, Technology, Engineering and Math (STEM) program to categorize the 15,283 DEGs that were differentially expressed in E13 vs. E18, E18 vs. E28, and E13 vs. E28 into eight possible expression profiles (*p*-value < 0.05) (Figure 5 and Table S10). The eight profiles were categorized into four different model profiles: (A) the consistent up-or down-regulation of genes over time in E13, E18, and E28 indicated that DEGs may contribute to stimulatory or inhibitory functions during the feather follicle initiation and development (profiles #7 and #0, Figure 6A), such as *WNT3A*, *IL20RA*, *BMP7*, *ASIP*, *LEF1*, *BRINP1*, *MSX1*, *TNFSF4*, *GATA3*, *NFATC1*, *IL-1R1*, *FGF1*, *WNT6*, *SHH*, *WNT5B*, *TRPS1*, *LHX6*, and *IL6R.* (B) the up-regulated or down-regulated genes only between E13 and E18 revealed that these DEGs merely played a significant role in development before secondary feather follicles initiation (profiles #6 and #1, Figure 6B), such as *TCF21*, *FGF12*, *SOX17a*, *TCF15*, *WNT2*, *NFATC2*, *FOXN4*, *TNFRSF13B*, *LRRC3B*, *IL17D*, *TNFSF10*, *PDGFB*, *TNFSF15*, *SOX17*, *TNFSF11*, and *IL15RA.* (C) the up-regulated or down-regulated genes only between E18 and E28 indicating that these DEGs could only contribute in growth after secondary feather follicles and these DEGs could participate functionally in development before secondary feather follicles initiation (profiles #4 and #3, Figure 6C), such as *IGFBP3*, *TCF7*, *TLE1*, *SOX8*, *SOX11*, *PDGFD*, *WNT3*, *WNT9B*, *KRT18*, and *SFRP2*, and (D) from E13 to E18 there was gene up-regulation and followed the down-regulation from E18 to E28 or genes were down-regulated from E13 to E18 and followed the up-regulation from E18 to E28, showing that these DEGs may play a stimulatory action in development before secondary feather follicles initiation and an inhibitory activity in development after secondary feather follicles initiation or vice versa (profiles #5 and #2, Figure 6D), such as *SOX10*, *LRRC20*, and *WNT2B.*

### 2.7. Validation of RNA-Sequencing (RNA-Seq) Data Using Quantitative Real-Time PCR (qRT-PCR)

**Figure 7:**
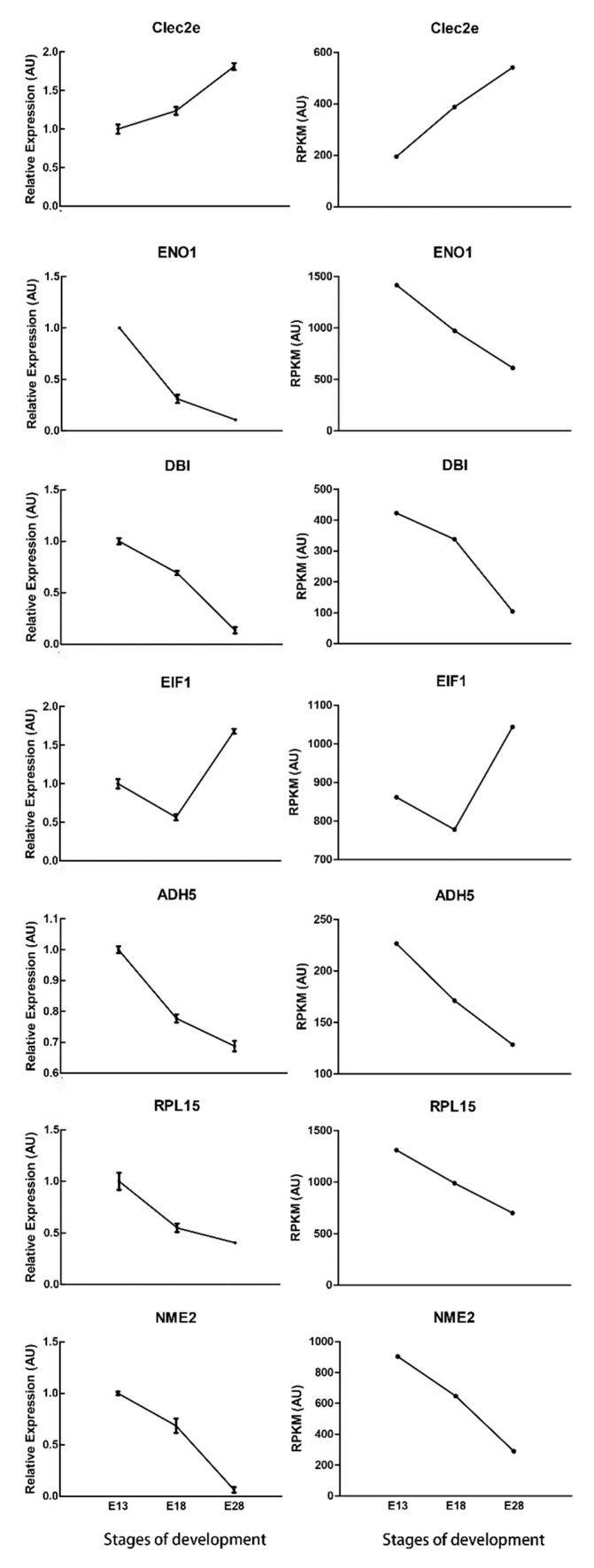
Quantitative Real-Time PCR (qRT-PCR) validation of DEGs at different stages of development. The left side indicates the data from qRT-PCR, which are shown as means ± standard error (SE) of three replicates; the right side shows reads per kb per million reads (RPKM) from RNA- Sequencing (RNA-seq), DEGs are defined as False Discovery Rate (FDR) < 0.05 and |log_2_^Fold Change^| > 1.

Quantitative Real-Time PCR (qRT-PCR) was used to validate the RNA-Seq data based on the transcript levels of seven DEGs: *Clec2e* (C-type lectin domain family 2, member e), *ENO1* (enolase 1), *DBI* (diazepam binding inhibitor), *EIF1* (eukaryotic translation initiation factor 1), *ADH5* (alcohol dehydrogenase 5), *RPL15* (ribosomal protein L15), and *NME2* (NME/NM23 nucleoside diphosphate kinase 2), which were selected randomly based on the expression profiles in three developmental stages. Combining with the cluster analysis, *ENO1* and *NME2* both belonged to the profile #0 with the consistent down regulation among E13, E18, and E28, and *Clec2e* was in the profile #7 with the consistent up regulation, which indicated that *ENO1*, *NME2*, and *Clec2e* may contribute to the inhibitory or stimulatory functions during the skin and feather follicle development. *DBI* was categorized into the profiles #3, which was down regulated only between E18 and E28, indicating that *DBI* might play an important role in the development of secondary feather follicles. *EIF1*, *ADH5*, and *RPL15* were enriched in GO terms cellular biosynthetic process, metabolic process, and organic substance biosynthetic process, respectively. All of the selected DEGs were successfully amplified with single bands with the expected sizes and the genes expression levels detected by qRT-PCR correlated with the RNA-Seq data (Figure 7), confirming the reliability and precision of the RNA-Seq data.

## 3. Discussion

Regardless of the similarities in structure, some functional differences between mammalian skin and hair, and avian skin and feathers between contour and downy feathers exist [19]. The transcriptome analysis of skin in the course of time (E13, E18, and E28) aided us to cross-examine the dynamic changes in comprehensive gene expression during goose embryonic development. We may perhaps study arrays of gene expression in embryo skin tissue at various stages of development for each gene to reconstruct the complete developmental expression pattern by gene expression profiling. In this study, we observed that the embryonic periods E13, E18, and E28 reflect the primordial period of primary feather follicles, primordial period of secondary feather follicles, and greater developmental period of secondary feather follicles in geese (*Anser anser*). This division of embryonic developmental periods could provide a fundamental knowledge into the biological processes that are involved in goose feather development. This is the first study relating goose (*Anser anser*) genome-extensive transcriptome analysis and histological changes to unveil the genes regulating feather follicles initiation and development that can provide new perception into the molecular components of feather follicles development.

Skin is composed of three layers: epidermis, dermis, and subcutaneous tissue [20]. Epidermal keratinocytes are known to synthesizing various cytokine proteins, including interleukins (ILs) [21]. According to a series of RNA-seq analysis, we identified 29 DEGs belonging to the ILs. The action of ILs can mediate inflammation and immunity, and it can influence differentiation, survival, and cell proliferation as non-immune responses within the tissues, thus playing important role in tissue restoration and wound recovery [22]. Interleukin-20-receptor I complex (*IL-20-RI*) is a member of the ILs family comprising of two strings: *IL20RA* and *IL20RB* [23]. Sa et al. [24] reported that *IL20RA* and *IL20RB* were highly expressed in the skin, and the overexpression of interleukin 20 (*IL-20*) induced conditions in the skin congruent to psoriasis. Feathers comprise of complicated well-organized dehydrated dead cells with keratin, and keratin synthesis can quickly develop as the key product of the synthetic actions of feather cells [25]. *IL-20* can promote hyper proliferation, and aberrant keratinocytes differentiation, which are the distinctive characteristics of psoriasis [26]. For our results, combined with the model profile (A), *IL20RA* was down-regulated in E13 vs. E18, E18 vs. E28 and E13 vs. E28, these results suggest that *IL20RA* might promote normal cell proliferation and differentiation at each stage of embryonic feather follicle development. Interleukin-6 receptor (*IL6R*) has been reported to have important contributory effect in asthma treatment [27] and have a potential role against pathogenic infection mastitis [28]. In this study, the results showed *IL6R* in model profile (A) was up-regulated, which suggests that *IL6R* may possess the protective functional role in feather follicle growth and development. Similar to the current study, the research of Ferreira et al. [29] showed that *IL6R* played the partial role in inflammatory disease inhibition during hair growth. Interleukin-1 (*IL1*) is a pro-inflammatory cytokine and the *IL1* system has been identified in mammalian reproduction system and in embryos [30]. Interleukin-1 type I receptor (*IL-1R1*) is an interleukin cytokine receptor; the gene encoding this protein is a member of the interleukin-1 receptor family [31]. Liao et al. [32] reported that *IL-1R1* gene in yellow bonefish (*Pelteobagrus fulvidraco*) might play primary activities during the growth stages of embryonic development through the ontogenetic expression analyses. Maurer et al. [33] reported that the functional role of the *IL-1* receptor in the epithelial-mesenchymal interactions was associated with the induction of hair follicle development. The RNA-seq showed that *IL-1R1* included in model profile (A) was up-regulated over time in E13, E18, and E28, indicating that goose embryo immune system was enhanced throughout the feather follicle development stages.

The primary functions of the DEGs can be elucidated by the GO analysis, and the main pathway where the DEGs are involved can be identified by the KEGG pathway analysis [34]. Previous study conducted by Chu et al. [35] indicated that *WNT3A*, *BMP7*, and *SFRP2* are differentially expressed in feather follicle tissue regeneration with both *BMP7* and *SFRP2* predominantly expressed in dermal papillae (DP), while *WNT3A* was decreased in the ramogenic zone epithelium (Erz). DEGs (*WNT3A*, *IGFBP3*, *BMP7*, and *SFRP2*) were enriched in GO terms, which are related to regulation of developmental growth, developmental process, organ morphogenesis, and embryonic appendage morphogenesis. Wnt pathway is one of the key signal mechanisms related to cell differentiation during embryogenesis, hematopoiesis, and carcinogenesis [36]. Kishimoto et al. [37] established that *WNT3A* as inductive signals maintained the mouse dermal papilla (DP) in an anagen state. Yue et al. [38] found that *WNT3A* was predominantly expressed in the anterior/rachis side, but it had none or lower expression in the posterior barb generative zone. The results in model profile (A) showed that *WNT3A* was down-regulated over time in E13, E18, and E28; suggesting that *WNT3A* was expressed in embryonic feather follicle development in a stage-dependent manner with a decreasing role towards feather follicle maturation. The secreted frizzled-related protein (*SFRP*) family consists of five different glycoproteins (*SFRP1-5*), each containing a highly homologous cysteine-rich domain and putative binding site on the frizzled receptor binding with *Wnt* ligands consequently acting as an extracellular antagonists and inhibitory factor 1 of *Wnt* [39,40]. However, Kwack et al. [41] found that *SFRP2* increased *WNT3A*-mediated catenin signaling in human dermal papilla cells and Kim et al. [42] reported that *SFRP2* might play a role in the catagen phase by inhibiting the proliferation of keratinocyte. We found that *SFRP2* in model profile (C) was only down-regulated between E18 and E28, which was the secondary stage for feather follicles development, suggesting that *SFRP2* might play a regulatory role in various phases of feather follicle development. Bone morphogenetic protein (BMP) signalling is involved in major processes during embryonic development and adult tissues by regulating the key cellular components of the skin, including hair follicle and epidermal keratinocytes, melanocytes, dermal fibroblasts, and epithelial stem cells [43,44]. *BMP7* is a member of the bone morphogenetic protein (BMP) family, which plays important functions in forming and patterning ventral and posterior tissues during vertebrate embryogenesis, and anti-fibrotic role [45,46]. Harris et al. [47] demonstrated *BMP7* is an early molecular marker for epidermal organ development with the feathers and scales development in a chick. Our study revealed that *BMP7* in model profile (A) was down-regulated, and thus predominantly expressed in the primary feather follicles. Herein, our data demonstrated that *BMP7* might significantly exert the stimulatory effect on the cell proliferation at the initial stage of embryonic feather follicle development. Insulin-like growth factor binding protein-3 (*IGFBP-3*) is one of a family of six homologous proteins (an N-linked glycosylated, phosphorylated, secretory protein) that bind insulin like growth factor 1 (*IGF-1*) and insulin like growth factor 2 (*IGF-2*) with high affinity, and have been implicated as both positive and negative regulators in cell growth and cell death in a context-dependent manner [48,49]. In this study, *IGFBP3* in model profile (C) was up-regulated between E18 and E28, which means that *IGFBP3* may play innumerable involvement in the development and maturity of feather follicles at E18 and E28 stage. The study by Weger et al. [50] in hair follicle expression of *IGFBP3* caused hypoplasia in the epidermis without any effects on the differentiation program, and *IGFBP3* expression alone could not alter the progression through the hair cycle. RNA-seq can help us to predict the DEGs, and these DEGs need further study to identify their functions in feather follicles and skin development.

To evaluate the differentially expressed genes in different embryonic follicle development stages, further analysis of the KEGG signaling pathways were carried out. The main pathways anticipated in the current study were documented in previous studies. ECM-receptor interaction, cell adhesion molecules (CAMs), cytokine-cytokine receptor interaction, glycine, serine and threonine metabolism, focal adhesion, neuroactive ligand-receptor interaction, tyrosine metabolism, steroid hormone biosynthesis, melanogenesis, Hedgehog signaling pathway, and phagosome were the major signaling pathways, which were all amongst the top 20 enriched pathways in all the three stages regulating embryonic feather formation. The majority of these signaling pathways are known to regulate various cell biological processes, such as in morphogenesis and the maintenance of structure and function, cell proliferation and survival [51,52], and skin and hair follicle development [53,54]. Conversely, with the exemption of the commonly known signaling pathways in all the three stages, there existed other signaling pathways that were significantly (*p* ˂ 0.05) enriched per the stage of development including arachidonic acid metabolism (E13 vs. E18), tight junction (E18 vs. E28), and DNA replication (E13 vs. E28). These pathways are regarded to be essential for the cellular process, genetic information processing, and lipid metabolism. Thus, we recommend that the expression and function of genes that are involved in these signaling pathways could be liable for feather quantity and quality.

Finally, we selected seven DEGs (*Clec2e*, *ENO1*, *DBI*, *EIF1*, *ADH5*, *RPL15,* and *NME2*) to validate the RNA-Seq data using qRT-PCR. The accuracy and consistency of RNA-Seq data were validated by the results of qRT-PCR. Moreover, we found that diazepam binding inhibitor (*DBI*) was down-regulated in both qRT-PCR and RNA-seq result. Alho et al. [55] reported that *DBI* immunoreactivity was found in the epidermis, eccrine sweat, and sebaceous glands. In this study, the GO analysis identified that *DBI* was related to epithelium development and epidermis development. Furthermore, the physiologic role of *DBI* in goose feather follicles and skin needs more study.

## 4. Materials and Methods

### 4.1. Ethics Statement

Goose embryos were slaughtered by cervical dislocation. All animal experiments were carried out by relevant guidelines that were developed by the China Council on Animal. All animal experimental procedures were approved by Goose Industry Research and Development Center of Jilin Agricultural University (Approval number: GR(J)18-006. Date: 29 March 2018).

### 4.2. Animal Sample Preparation

In total, 200 embryos of geese (*Anser anser*) in a core breeding group (Jilin Agricultural University, Jilin Province, Northeast of China) were incubated in one incubator according to the routine procedure. During the incubation, we randomly selected three eggs every day from the tenth day to the twenty-eighth days for observation. We observed the feather placodes on the dorsal tracts had started to protrude, whereas those of the other tracts had not, and no feather placodes were observed in the midline of the ventral tracts, while towards to the legs, the diameter of feather placode became smaller at the embryonic day (E13), deducing that it was the primordial period of primary feather follicles; At the embryonic day (E18), the feather placode displayed an uneven pattern on the skin surface and the dorsal feather began keratinization, we deduced that it was the primordial period of the secondary feather follicles. At the embryonic day (E28), the body surface of the embryonic goose was covered with yellow and black lanugos; we deduced that it is the greater developmental period of the secondary feather follicles [56].

At this time, six embryos were randomly selected at each stage (E13, E18, and E28), respectively. We sampled the cross region (about 1 cm^2^) of the midline and two wings of dorsal skin. For each stage, three samples selected for total RNA extraction were cryopreserved at −80 °C after being frozen in liquid nitrogen immediately and the other remaining nine samples were fixed in 10% phosphate-buffered formalin for tissue sectioning.

### 4.3. Histological Observation

Fixing the skin samples into the 10% formalin for 24 h, then washing them with running tap water overnight, and the samples were dehydrated in increasing grades of ethanol: 70% alcohol for 30 min, 80% alcohol for 6 h, 90% alcohol for 6 h, 95% alcohol for 6 h, and 100% alcohol two times for 6 h each. Later, a KD-BM tissue embedding processor (JinhuaKedi Instrumental Equipment Co., Ltd., Jihua city, Zhejiang, China) was used to do the paraffin embedding at 70 °C. The tissues were sliced by Leica RM 2135 microtome (Leica Microsystems, Wetzlar, Germany) and the thickness of the slices was 5 μm. After the paraffin sections were dewaxed and hydrated, staining was performed by Hematoxylin and Eosin (H&E) staining and mounting with neutral balsam. All of the slides were observed under a Nicon-300 light microscope (Nicon, Tokyo, Japan).

### 4.4. RNA Extraction, Library Preparation, and Sequencing

Total RNA of the dorsal skin tissues from the E13, E18, and E28 goose embryos was extracted using the TRIzol Reagent (Invitrogen Life Technologies, Carlsbad, CA, USA) following the manufacturer’s protocol NanoDrop 2000 (Thermo Scientific, Wilmington, DE, USA) and Agilent 2100 (Agilent Technologies, Santa Clara, CA, USA) instruments were used to evaluate the concentration and quality of the total RNA. RNA samples used in the preceding analysis had RNA integrity number (RIN) ≥ 8.4, 28S/18S ≥ 1.0, 1.8 < OD260/280 < 2.0.

To construct the reference transcriptome assembly library, the total RNA from the three selected samples at each stage was used. Poly (A) mRNA was purified from total RNA samples with oligo(dT) cellulose. Continually, the first-strand cDNA was generated from mRNA fragments and reverse transcribed randomly by primers, while DNA polymerase I and RNase H were used to synthesize the second-strand cDNA (NEBNext Ultra RNA Library Prep Kit (NEB#E7530)). Hereafter, QiaQuick PCR extraction kit (Qiagen, Hilden, Germany) was used to purify the double-strand cDNAs, which were subjected to end pairing, the addition of a single “A” bases, and ligated to Illumina adapters. Agarose gel electrophoresis and PCR amplification were used to make the ligation products size fractioned and the fragments enrichment, respectively. Finally, the amplified fragments were sequenced using Illumina HiSeq™ 4000 by Gene Denovo Co. (Guangzhou, China), according to the manufacturer’s instructions. The high quality Illumina sequencing data have been submitted in the National Center for Biotechnology Information (NCBI) sequence read archive (SRA) database with access number SRP156879.

### 4.5. De Novo Assembly and Gene Annotation

For the assembly library, raw sequence transcripts in the cluster units were defined as unigenes. Data of the libraries were filtered to remove those containing adapter and reads with more than 10% unknown nucleotides, and reads with more than 50% of low-quality base (value ≤ 5). *De novo* assembly of the clean reads was carried out by Trinity software (version: 2.1.1, Broad Institute of Massachusetts Institute of Technology and Harvard, Cambridge, MA, USA), with default parameters and no reference sequence. The transcripts were further clustered and assembled according to nucleotide sequence identity. To eliminate redundant sequences, the longest transcripts in the cluster units were defined as unigenes.

To identify the putative functions of unigenes, a BLASTx search at NCBI with an *E*-value threshold of 1 × 10^−5^ to NCBI Nr protein database (available online: http://www.ncbi.nlm.nih.gov), Swiss-Prot protein database (available online: http://www.expasy.ch/sprot), KEGG pathway database (available online: http://www.genome.jp/kegg), and KOG database (available online: http://www.ncbi.nlm.nih.gov/COG) were used to perform functional annotation. According to the best alignment from the four databases, the sequence direction of the unigenes was assigned. When the results conflicted among databases, prioritizing in the order Nr, Swiss-Prot, KEGG, and KOG. Blast2GO software package and BLASTall software against the KEGG database were used to identify the Gene Ontology (GO) (available online: http://www.geneontology.org/) annotation and KEGG pathway annotation, respectively. In addition, we used WEGO software (available online: http://wego.genomics.org.cn/cgibin/wego/index.pl) to perform the functional classification of unigenes.

### 4.6. Expression Annotation

The SOAPaligner/soap2 software was used to map clean reads into the transcriptome reference database. Not more than two mismatch bases were permitted, and unique mapped reads were obtained. The tag frequencies in the different digital gene expression (DGE) libraries were statistically analyzed to compare the differences in gene expression at different developmental stages. We calculated and normalized the number of unique-match reads to RPKM (reads per kb per million reads) for gene expression analysis. Expression difference of each gene between the three groups was compared by using edgeR (version: 3.16.5, Department of Medical Biology, the University of Melbourne, Parkville, VIC 3010, Australia). The *p* value corresponded to differential gene expression at statistically significant levels. We used FDR (False Discovery Rate) to determine the *p* value threshold. DEGs were defined as FDR < 0.05 and |log_2_^Fold Change^| > 1. Gene expression data were normalized to log_2_^(E18/E13)^, log_2_^(E28/E18)^, and log_2_^(E28/E13)^. DEGs were clustered by the STEM (Short Time-series Expression Miner, version: 1.3.8, Center for Automated and Learning and Discovery, School of Computer Science, Carnegie Mellon University, Pittsburgh, PA 15213, USA) [57]. We considered the clustered profiles with *p*-value < 0.05, as significantly expressed.

### 4.7. Validation of RNA-Seq Data by qRT-PCR

The procedure followed was previously described by Lu et al. [58] with modifications. Seven DEGs (*Clec2e*, *ENO1*, *DBI*, *EIF1*, *ADH5*, *RPL15*, and *NME2*) were selected and the expression profiles were validated by quantitative Real-Time PCR (qRT-PCR) to confirm the transcriptome data. First-strand cDNA was generated from 1 μg total RNA isolated from each of the skin samples using the Superscript first-strand synthesis system (Invitrogen, Shanghai, China). Primers for qRT-PCR were designed by using Primer3 Input (version: 0.4.0, available online: http://bioinfo.ut.ee/primer3-0.4.0/) and synthesized by Sangon Biotech (Shanghai Co., Ltd., Shanghai, China). β-actin was selected as an internal control for normalization. All of the primers are shown in Table S11. SYBR Green Realtime PCR Master Mix (TOYOBO, Osaka, Japan) was used to perform the qPCR reactions in Applied Biosystems 7500 Real-Time PCR System (Thermo Fisher Scientific Inc., Waltham, MA, USA). Each reaction mixture was 20 μL containing 10 μL of SYBR Green Real-time PCR Master Mix, 0.8 μL each of forward and reverse primers, 2 μL of cDNA, and 6.4 μL of distilled water. The qRT-PCR condition was 95 °C for 60 s; followed by 40 cycles of 95 °C for 15 s, 60 °C for 15 s, and 72 °C for 45 s; and, completed with a final stage of melting curve analysis. Each qPCR analysis was detected in triplicate. Relative quantification of gene expression levels was calculated by the 2^−ΔΔ*C*t^ method [59].

## 5. Conclusions

In the present study, H&E staining was used to observe histological changes, and *de novo* transcriptome analysis of the development of feather follicles in goose (*Anser anser*) was performed for the first time to establish dynamic transcriptome profiles at three stages (E13, E18, and E28). Subsequently, bioinformatics analyses (GO, KEGG, and series cluster) helped us to identify the key regulatory genes (*ILs*, *WNT3A*, and *IGFBP3*, among others) related feather follicles development, which suggests an important role for transcriptome analysis in the development of feather follicles and the increment of the down yield. This study does not only provide the useful transcriptomic reference for goose feather follicles, but also a benchmark to discover biomarkers that are associated to down product.

## Figures and Tables

**Table 1:** Statistics of data filtering.

Sample	Before Filter Reads Number	After Filter Reads Number (%)	Reads Len	GC	Adapter (%)	Low Quality (%)
E13-1	51,704,342	50,591,630 (97.85%)	150	57.45%	237,982 (0.46%)	869,940 (1.68%)
E13-2	53,263,218	52,209,704 (98.02%)	150	56.54%	226,048 (0.42%)	822,340 (1.54%)
E13-3	52,416,882	51,381,802 (98.03%)	150	56.84%	227,124 (0.43%)	803,064 (1.53%)
E18-1	51,066,402	50,087,188 (98.08%)	150	56.80%	212,366 (0.42%)	762,056 (1.49%)
E18-2	55,460,086	54,329,966 (97.96%)	150	56.78%	255,134 (0.46%)	869,672 (1.57%)
E18-3	50,868,330	49,791,358 (97.88%)	150	57.04%	231,472 (0.46%)	840,530 (1.65%)
E28-1	49,794,350	48,680,268 (97.76%)	150	56.43%	221,302 (0.44%)	888,292 (1.78%)
E28-2	48,238,510	47,164,092 (97.77%)	150	57.06%	234,242 (0.49%)	835,516 (1.73%)
E28-3	72,214,054	69,719,918 (96.55%)	150	56.56%	295,872 (0.41%)	2,192,284 (3.04%)
Total	485,026,174	473,955,926(97.72%)			2,141,542 (0.44%)	8,883,694 (1.83%)

**Table 2:** List of differentially expressed genes (DEGs) belonging to interleukins (ILs) family expressed in E13, E18, and E28, which were detectable in the feather follicles of goose.

Gene_ID	Gene_Symbol	RPKM ^1^-E13	RPKM-E18	RPKM-E28
Unigene0004079	*IL16*	0.313533	0.810267	1.96877
Unigene0004463	*IL20RA*	9.75107	3.5371	1.67913
Unigene0009028	*IL36RN*	0.7632	3.4918	12.3211
Unigene0029291	*IL17D*	0.470633	1.89013	2.22343
Unigene0033163	*ILDR2*	4.27267	10.8527	10.6716
Unigene0036855	*Il15ra*	1.66583	3.69263	4.09673
Unigene0008165	*IL7R*	0.001	0.0513667	1.09823
Unigene0016840	*IL17C*	3.03347	1.74543	0.0239
Unigene0022008	*IL31RA*	0.230633	0.4919	1.37237
Unigene0022142	*Il34*	0.631367	0.951633	2.5645
Unigene0025806	*IL16*	0.248633	0.353767	1.08887
Unigene0028720	*IL22RA1*	2.47243	1.55193	0.228967
Unigene0028801	*IL1RAP*	16.0848	16.0816	6.3824
Unigene0030418	*Il1rl1*	0.0184333	0.132833	0.776233
Unigene0030419	*Il1rl1*	0.2798	0.466433	0.1425
Unigene0031991	*IL13RA2*	0.934333	0.863233	8.49547
Unigene0036009	*IL12RB2*	1.8168	1.71347	0.647733
Unigene0038243	*IL1R1*	1.13113	2.188	6.5063
Unigene0041147	*Il15ra*	0.0634	0.0751667	1.1632
Unigene0001585	*IL1RN*	9.347	18.6492	24.2625
Unigene0008166	*IL7R*	0.001	0.145	0.9396
Unigene0017728	*IL10RB*	0.511767	1.11633	1.19017
Unigene0022006	*Il31ra*	0.0695667	0.252933	1.08303
Unigene0027228	*IL1RL2*	1.91883	1.12357	0.711267
Unigene0028870	*IL11RA*	21.1164	30.1148	50.5107
Unigene0030116	*IL17RC*	14.7142	21.909	40.9831
Unigene0030288	*IL2RG*	6.4065	11.2284	16.348
Unigene0031047	*IL6R*	14.2714	20.6134	39.1989
Unigene0052404	*IL16*	0.100367	0.320633	0.8613

^1^ RPKM: Reads Per Kilobase per Million mapped reads.

## Supplementary Materials

Supplementary materials can be found at http://www.mdpi.com/1422-0067/19/10/3170/s1.

## Abbreviations

E13	embryonic day 13
E18	embryonic day 18
E28	embryonic day 28
DEGs	differentially expressed genes
H&E	Hematoxylin and Eosin
RPKM	reads per kb per million reads
RNA-Seq	RNA-Sequencing
FDR	False Discovery Rate
WNT3A	Wnt family member 3A
IGFBP3	insulin-like growth factor binding protein 3
BMP7	bone morphogenetic protein 7
SFRP2	secreted-frizzled related protein 2
ILs	interleukins
IL-20-RI	Interleukin-20-receptor I complex
IL6R	Interleukin-6 receptor
IL-1R1	Interleukin-1 type I receptor
DBI	diazepam binding inhibitor
